# Type specimens of centipedes (Myriapoda, Chilopoda) in the National Museum, Prague (Czech Republic)

**DOI:** 10.3897/zookeys.510.8636

**Published:** 2015-06-30

**Authors:** Petr Dolejš

**Affiliations:** 1 Department of Zoology, National Museum-Natural History Museum, Cirkusová 1740, CZ-193 00, Praha 9-Horní Počernice, Czech Republic

**Keywords:** Zoological collection, Luděk Jindřich Dobroruka, *Allothereua*, *Lithobius*, *Pachymerium*, *Scolopendra*, *Strigamia*

## Abstract

The centipede collection in the National Museum in Prague contains type material of 16 taxa (14 species and two subspecies), of which 15 were described by Luděk J. Dobroruka and one by Karl W. Verhoeff: *Allothereua wilsonae* Dobroruka, 1979; *Chinobius alenae* Dobroruka, 1980; *Lithobius corrigendus* Dobroruka, 1988; *Lithobius creticus* Dobroruka, 1977; *Lithobius erythrocephalus mohelensis* Dobroruka, 1959; *Lithobius evae* Dobroruka, 1958; *Lithobius magurensis* Dobroruka, 1971; *Lithobius purkynei* Dobroruka, 1957; *Lithobius tatricus* Dobroruka, 1958; *Lithobius tatricus monounguis* Dobroruka, 1958; *Monotarsobius homolaci* Dobroruka, 1971; *Monotarsobius krali* Dobroruka, 1979; *Pachymerium dilottiae* Dobroruka, 1976; *Pachymerium hanzaki* Dobroruka, 1976; *Scolopendra aztecorum* Verhoeff, 1934 and *Strigamia olympica* Dobroruka, 1977. Of these 16 taxa, five were described from the Czech Republic, three from Slovakia and eight from other countries (Greece, Iraq, Kyrgyzstan, Mexico, Nepal, Russia and Uzbekistan). The eight taxa described from the Czech and Slovak Republics are now considered as junior synonyms but the eight taxa described from the other countries are still valid.

## Introduction

The most important part of the zoological collection of National Museum in Prague (NMP) is the type material. Up to now, catalogues of the type material of spiders (Růžička et al. 2005), vertebrates (Mlíkovský et al. 2011) and horsehair worms (Dolejš 2012) were published. The process of cataloguing the zoological type material is ongoing with this catalogue of centipede type material. There are no types of other myriapod classes despite 19 millipede and two symphylan taxa were described from the territory of the Czech Republic (Bezděk 2011). The centipede type material was deposited to the NMP in three distinct periods: 1930–1936 (material collected during expeditions organized by the NMP and people who were collaborating with the NMP), 1975–1978 (material collected by various collectors and provided to L. J. Dobroruka) and 2012–2014 (material found in Dobroruka’s personal collection). Majority of the material was collected by Czechs and the new taxa originated from these collections were described by L. J. Dobroruka and K. W. Verhoeff. The latter author described new *Scolopendra* species based on material collected by Czech acarologist Prof. Jaroslav Štorkán (20 April 1890 – 1 June 1942) who was collecting for the NMP in 1920s and 1930s. Thus, it is not surprising that a part of material collected by him came back to the NMP.

The collection of centipedes (containing ca. 1500 specimens representing ca. 150 species and subspecies from about 40 countries) is perfectly organized and catalogued thanks to RNDr. Ing. Luděk Jindřich Dobroruka (20 October 1933 – 4 July 2004) who worked in the NMP as a curator of Invertebrates in 1956 and continued with his work on centipedes in the NMP also in 1970s. His work resulted in publishing of catalogues of non-type Bulgarian (Dobroruka 1977a), Greek (Dobroruka 1977b) and Brazilian (Dobroruka 1979a) centipedes deposited there. Despite collaborating with the NMP, some types described by him remained in his personal collection and a part of them were deposited to the NMP after the work of Tuf et al. (2008). Unfortunately, the destination of several Dobroruka’s types is still unknown. More information about L. J. Dobroruka can be found in Felix (2004a, b), Hanák (2004), Kellnerová (2004), Růžička (2005), Tuf (2005) and Bartoš (2006).

## Methods

All the specimens are preserved in 80% ethanol. Nomenclatural issues follow the International Code of Zoological Nomenclature (ICZN 1999, 2012). Orig. = original combination (as proposed in the original publication), the first page with descriptions including figures and sex of the illustrated animal. Now = current status of the taxon. For grid squares of Czech and Slovak localities, see Buchar (1982) and Pruner and Míka (1996). Condition of the type material is provided as follows: excellent – good – reasonable – poor.

## Results

**Order: Scutigeromorpha**

**Family: Scutigeridae**

***Allothereua wilsonae* Dobroruka, 1979**

**Orig.** *Allothereua wilsonae* Dobroruka, 1979b: 101, figs 1–5 (♀).

**Now.** *Allothereua wilsonae* Dobroruka, 1979. The status of this species has never been revised since its original description.

**Holotype.** NMP P6E-1761, ♀ in poor condition (in two pieces, legs detached), collected by Jane M. Wilson on 14 October 1971 in one of the tents of the camp, near Mahendra Cave, Pokhara Valley, Nepal; 28°14.00'N, 83°59.00'E.

**Order: Scolopendromorpha**

**Family: Scolopendridae**

***Scolopendra aztecorum* Verhoeff, 1934**

**Orig.** *Scolopendra aztecorum* Verhoeff, 1934: 49.

**Now.** *Scolopendra aztecorum* Verhoeff, 1934. Valid species according to Cupul-Magaña (2013).

**Syntype.** NMP P6E-1303, one adult specimen in good condition, collected by Jaroslav Štorkán in 1930 under decaying cactuses, La Paz, Baja California Sur Region, Mexico; 24°8.28'N, 110°18.58'W.

**Note.** Other syntypes are deposited in the Zoologisches Museum der Humboldt-Universität, Berlin, Germany under number ZMB 13378 (Moritz and Fischer 1979); in the Bavarian State collection, Munich, Germany under numbers ZSM/Myr-20051044 and ZSM/Myr-20051045 (SysTax 2015); and in the Naturalis Biodiversity Center, Leiden, the Netherlands under number RMNH.CHIL.154 (C. A. Martínez-Muñoz in litt.). Both Shelley (2006) and Cupul-Magaña (2013) erroneously regarded the specimen from Berlin as a holotype despite Verhoeff (1934) did not designate any type specimen and despite Moritz and Fischer (1979) referred to it as “Syntypus”.

**Order: Geophilomorpha**

**Family: Geophilidae**

***Pachymerium dilottiae* Dobroruka, 1976**

**Orig.** *Pachymerium dilottiae* Dobroruka, 1976: 259, figs 1A–D (♂♀).

**Now.** *Pachymerium dilottiae* Dobroruka, 1976. The status of this species has never been revised since its original description.

**Holotype.** NMP P6E-1326, ♀ in reasonable condition (in two pieces, head mounted separately on a permanent slide), collected by Vlasta Kálalová-di Lotti in 1929 in Baghdad, Iraq; 33°20.43'N, 44°24.05'E.

**Paratypes.** NMP P6E-1327, 1♂ in good condition and 1♂ in reasonable condition (missing the caudal part of the body), same data as holotype.

***Pachymerium hanzaki* Dobroruka, 1976**

**Orig.** *Pachymerium hanzaki* Dobroruka, 1976: 260, figs 2A–E (♂).

**Now.** *Pachymerium hanzaki* Dobroruka, 1976. The status of this species has never been revised since its original description.

**Holotype.** NMP P6E-1328, ♂ in reasonable condition (head mounted separately on a permanent slide), collected by Vlasta Kálalová-di Lotti in 1929 in Baghdad, Iraq; 33°20.43'N, 44°24.05'E.

**Family: Linotaeniidae**

***Strigamia olympica* Dobroruka, 1977**

**Orig.** Strigamia (Strigamia) olympica Dobroruka, 1977b: 163, figs 5–8 (♀).

**Now.** *Strigamia olympica* Dobroruka, 1977. Valid species according to Bonato et al. (2012).

**Holotype.** NMP P6E-1352, ♀ in reasonable condition, collected by Karel Táborský on 5 June 1935 in Óros Ólympos Mt., Greece; 40°3.72'N, 22°20.70'E.

**Order: Lithobiomorpha**

**Family: Lithobiidae**

**Lithobius (Chinobius) alenae (Dobroruka, 1980)**

**Orig.** *Chinobius alenae* Dobroruka, 1980: 92, figs 1–6 (♀).

**Now.** Lithobius (Chinobius) alenae (Dobroruka, 1980). The status of this species has never been revised since its original description.

**Holotype.** NMP P6E-3912, ♀ in reasonable condition, collected by Alena Čepická on 5 July 1977 under bark of fallen trees in Voronezhskove, 30 km NE from Khabarovsk, Khabarovskiy Kray, Russia; 48°41.67'N, 135°13.20'E.

**Paratype.** NMP P6E-3913, ♀ in good condition, same data as holotype.

**Lithobius (Lithobius) corrigendus Dobroruka, 1988**

**Orig.** *Lithobius corrgendus* (sic!) Dobroruka, 1988: 6 (*nomen novum* for *Lithobius parvus* Folkmanová, 1946).

**Now.** Junior synonym of Lithobius (Lithobius) lucifugus L. Koch, 1862 (Tuf et al. 2008).

**Neotype.** NMP P6E-3901, ♂ in reasonable condition, collected by Luděk J. Dobroruka on 12 August 1997 on the left bank of the Dyje river by Devět mlýnů, Podyjí National Park, Znojmo District, Jihomoravský Region, Czech Republic; 48°49.08'N, 15°58.38'E (7161d); designated by Tuf et al. (2008).

**Note.** Another (non-type) female specimen in excellent condition (NMP P6E-3902) determined by Dobroruka as *Lithobius corrigendus* was revised as Lithobius (Lithobius) latro Meinert, 1872 by Jolanta Wytwer, Ivan H. Tuf and Karel Tajovský in 2006.

**Lithobius (Lithobius) creticus Dobroruka, 1977**

**Orig.** *Lithobius creticus* Dobroruka, 1977b: 161, figs 1–4 (♀).

**Now.** Lithobius (Lithobius) creticus Dobroruka, 1977. Valid species according to Zapparoli (2002).

**Holotype.** NMP P6E-1350, ♀ in good condition, collected by Josef Mařan and Otakar Štěpánek in 1937 in Óros Ídi Mt., Crete, Greece; 35°14.04'N, 24°46.84'E.

**Paratype.** NMP P6E-1351, ♂ in good condition, same data as holotype.

**Lithobius (Lithobius) erythrocephalus mohelensis Dobroruka, 1959**

**Orig.** *Lithobius erythrocephalus mohelensis* Dobroruka, 1959: 105, figs 3 (♂), 4 (♀).

**Now.** Lithobius (Lithobius) erythrocephalus C. L. Koch, 1847 (Tuf et al. 2008).

**Syntypes.** NMP P6E-3078, 2♂♂ and 1♀ in poor condition (legs detached), collected by Luděk J. Dobroruka in April and June 1955 in Serpentine Steppe of Mohelno, Třebíč District, Vysočina Region, Czech Republic; 49°6.47'N, 16°11.25'E (6863c).

**Lithobius (Lithobius) evae Dobroruka, 1958**

**Orig.** Lithobius (Lithobius) evae Dobroruka, 1958b: 26, figs 1 (♂), 2 (♀).

**Now.** Junior synonym of Lithobius (Lithobius) tenebrosus Meinert, 1872 (Tuf et al. 2008).

**Type material.** NMP P6E-3079, 2♂♂ and 4♀♀ in poor condition (legs detached), collected by Eva M. Homoláčová and Miloš E. Homoláč on 14 September 1956 among fallen beech leaves in Bukovec Hill near Jizerka, Jizerské hory Mts., Jablonec nad Nisou District, Liberec Region, Czech Republic; 50°48.86'N, 15°21.47'E (5158c).

**Note.** Male holotype and three female paratypes were not labelled and thus indistinguishable among the type material mentioned.

**Lithobius (Monotarsobius) homolaci Dobroruka, 1971**

**Orig.** *Monotarsobius homolaci* Dobroruka, 1971: 262, fig. 2 (♂).

**Now.** junior synonym of Lithobius (Sigibius) burzenlandicus Verhoeff, 1931 (Tuf et al. 2008).

**Holotype.** NMP P6E-1454, ♂ in good condition, collected (sifted from beech leaf litter) by Miloš E. Homoláč in September 1959 in Spišská Magura Mts., Kežmarok District, Prešov Region, Slovakia; 49°17.04'N, 20°22.38'E (6788a).

**Paratype.** NMP P6E-1455, ♀ in reasonable condition, same data as holotype.

**Lithobius (Monotarsobius) krali (Dobroruka, 1979)**

**Orig.** *Monotarsobius krali* Dobroruka, 1979c: 161, figs 1–3 (♂♀), 4 (♀).

**Now.** Lithobius (Monotarsobius) krali (Dobroruka, 1979). Valid species according to Dányi and Tuf (2012).

**Holotype.** NMP P6E-1762, ♂ in good condition, collected by Josef Král on 8 July 1976 in Ala Archa, Chüy Region, Kyrgyzstan; 42°38.70'N, 74°28.73'E.

**Paratype.** NMP P6E-1763, ♀ in reasonable condition, collected by Josef Král on 3 July 1976 in Gora Chimgan Mt., Toshkent Region, Uzbekistan; 41°40.45'N, 69°42.90'E.

**Lithobius (Lithobius) magurensis Dobroruka, 1971**

**Orig.** *Lithobius magurensis* Dobroruka, 1971: 261, fig. 1 (♂).

**Now.** Junior synonym of Lithobius (Lithobius) muticus C. L. Koch, 1847 (Tuf et al. 2008).

**Holotype.** NMP P6E-1452, ♂ in excellent condition, collected by Miloš E. Homoláč in September 1959 in Spišská Magura Mts., Kežmarok District, Prešov Region, Slovakia; 49°17.04'N, 20°22.38'E (6788a).

**Paratype.** NMP P6E-1453, ♀ in good condition, same data as holotype.

**Lithobius (Lithobius) purkynei Dobroruka, 1957**

**Orig.** Lithobius (Lithobius) purkyněi Dobroruka, 1957: 174, figs 1A–B (♀).

**Now.** Junior synonym of Lithobius (Lithobius) macilentus L. Koch, 1862 (Tuf et al. 2008).

**Type material.** NMP P6E-3080, 5♀♀ in poor condition (legs detached), collected by Luděk J. Dobroruka on 17 and 22 June 1956 in Boreč, Litoměřice District, Ústí nad Labem Region, Czech Republic; 50°30.85'N, 13°59.31'E (5449d).

**Note.** Female holotype and four female paratypes were not labelled and thus indistinguishable among the type material mentioned. Other (non-type) male and female specimens (NMP P6E-1290) determined by Dobroruka as *Lithobius purkynei* were mentioned in Dobroruka (1977c).

**Lithobius (Lithobius) tatricus Dobroruka, 1958**

**Orig.** Lithobius (Archilithobius) tatricus Dobroruka, 1958a: 115, figs 1-2 (♂♀), 3 (♀).

**Now.** Junior synonym of Lithobius (Lithobius) mutabilis L. Koch, 1862 (Tuf et al. 2008).

**Type material.** NMP P6E-3081, three specimens in a poor condition (legs detached), collected by Luděk J. Dobroruka on 26 June 1955 from sieved material and under bark of fallen trees in Bielovodská dolina Valley, Belanske Tatry Mts., Poprad District, Prešov Region, Slovakia; 49°13.31'N, 20°6.14'E (6786d).

**Note.** Male holotype, three male and one female paratypes were not labelled and thus indistinguishable among the type material mentioned. Other (non-type) male and female specimens (NMP P6E-1376, P6E-1380, P6E-1427, P6E-1439 and P6E-3082) determined by Dobroruka as *Lithobius tatricus* were mentioned in Dobroruka (1977c, 1998).

**Lithobius (Lithobius) tatricus monounguis Dobroruka, 1958**

**Orig.** Lithobius (Archilithobius) tatricus monounguis Dobroruka, 1958b: 27.

**Now.** Junior synonym of Lithobius (Lithobius) latro Meinert, 1872 (Tuf et al. 2008).

**Holotype.** NMP P6E-3082, ♂ in poor condition (legs detached), collected by Eva M. Homoláčová and Miloš E. Homoláč on 14 September 1956 under moist beech leaves in Bukovec Hill near Jizerka, Jizerské hory Mts., Jablonec nad Nisou District, Liberec Region, Czech Republic; 50°48.86'N, 15°21.47'E (5158c).

## Conclusions

The zoological collection of the National Museum in Prague hosts (among others) type material of 16 centipede taxa. Both sctutigeromophs (Scutigeridae) and scolopendromorphs (Scolopendridae) are represented by one species, geophilomorphs (Geophilidae and Linotaenidae) by three species and lithobiomorphs (Lithobiidae) by 11 species and subspecies. Five species from the first three orders (*Allothereua wilsonae*, *Scolopendra aztecorum*, *Pachymerium dilottiae*, *Pachymerium hanzaki* and *Strigamia olympica*) and three species from Lithobiomorpha (*Lithobius alenae*, *Lithobius creticus* and *Lithobius krali*) are currently valid. The eight remaining lithobiomorph species and subspecies (*Lithobius corrigendus*, *Lithobius erythrocephalus mohelensis*, *Lithobius evae*, *Lithobius homolaci*, *Lithobius magurensis*, *Lithobius purkynei*, *Lithobius tatricus* and *Lithobius tatricus monounguis*) are now considered as junior synonyms.
